# Amino Acids Regulate Transgene Expression in MDCK Cells

**DOI:** 10.1371/journal.pone.0096823

**Published:** 2014-05-05

**Authors:** Marta Torrente, Adriano Guetg, Jörn Oliver Sass, Lisa Arps, Lisa Ruckstuhl, Simone M. R. Camargo, François Verrey

**Affiliations:** 1 Institute of Physiology and Zurich Center of Integrative Human Physiology, University of Zurich, Zurich, Switzerland; 2 Division of Clinical Chemistry & Biochemistry, University Children's Hospital, Zurich, Zurich, Switzerland; University of Cambridge, United Kingdom

## Abstract

Gene expression and cell growth rely on the intracellular concentration of amino acids, which in metazoans depends on extracellular amino acid availability and transmembrane transport. To investigate the impact of extracellular amino acid concentrations on the expression of a concentrative amino acid transporter, we overexpressed the main kidney proximal tubule luminal neutral amino acid transporter B^0^AT1-collectrin (SLC6A19-TMEM27) in MDCK cell epithelia. Exogenously expressed proteins co-localized at the luminal membrane and mediated neutral amino acid uptake. However, the transgenes were lost over few cell culture passages. In contrast, the expression of a control transgene remained stable. To test whether this loss was due to inappropriately high amino acid uptake, freshly transduced MDCK cell lines were cultivated either with physiological amounts of amino acids or with the high concentration found in standard cell culture media. Expression of exogenous transporters was unaffected by physiological amino acid concentration in the media. Interestingly, mycoplasma infection resulted in a significant increase in transgene expression and correlated with the rapid metabolism of L-arginine. However, L-arginine metabolites were shown to play no role in transgene expression. In contrast, activation of the GCN2 pathway revealed by an increase in eIF2α phosphorylation may trigger transgene derepression. Taken together, high extracellular amino acid concentration provided by cell culture media appears to inhibit the constitutive expression of concentrative amino acid transporters whereas L-arginine depletion by mycoplasma induces the expression of transgenes possibly via stimulation of the GCN2 pathway.

## Introduction

Mammals have developed a finely tuned response to changes in nutrient availability. Amino acid sufficiency activates the mammalian target of rapamycin (mTOR) pathway, which ultimately promotes protein synthesis and cell growth. In contrast, amino acid limitation initiates an amino acid response (AAR) signaling cascade, which regulates multiple steps in gene expression including chromatin structure modification, transcription and translation [1]. Individual cells sense amino acid deficiency through an accumulation of uncharged tRNAs which bind to and activate the GCN2 protein kinase [2]–[4]. GCN2 protein kinase phosphorylates and inactivates the eukaryotic initiation factor 2α (eIF2α), which in turn leads to a decrease of global mRNA translation [5], [4]. To compensate for this restriction, the expression of a spectrum of genes involved in the adaptive response to nutritional stress is stimulated. This phenomenon, known as translational derepression, was first described for the yeast transcription factor GCN4 [6], and then shown for the mammalian GCN4 homologue, activating transcription factor 4 (ATF4) [7]. ATF4 binds to C/EBP-ATF response elements (CARE)-containing genes and triggers their transcription. Among the genes induced by amino acid limitation, amino acid transporters (*SLC7A1*, *SLC38A2*), transcription factors (*CHOP*) or amino acid metabolic enzymes (*ASNS*) have been identified (reviewed in Ref. [1]).

The transport of amino acids across epithelial cells is mediated by a wide array of membrane proteins. Previously, to investigate the role of specific amino acid transporters, we reconstructed the transepithelial exchange of L-Arg and L-Leu by expressing the luminal heterodimeric amino acid antiporter b^0,+^-rBAT (SLC7A9-SLC3A1) and the basolateral heterodimeric antiporters y^+^LAT1-4F2hc (SLC7A7-SLC3A2) and LAT2-4F2hc (SLC7A8-SLC3A2) in MDCK epithelial cells [8], [9]. This cell line of distal nephron origin represents a well-established model for epithelial polarity studies [10], [11]. The overall directional (re)absorptive transport of amino acids is however driven by accumulative transporters located in the luminal membrane of transporting epithelia. In the proximal kidney tubule and in the small intestine, this central role is mainly played by the luminal sodium symporter for neutral amino acids B^0^AT1 (SLC6A19) [12], [13]. Mutations in its gene have been shown to cause autosomal recessive Hartnup disorder [14], [15]. The expression and function of B^0^AT1 depends on the co-expression of members of the renin angiotensin system (RAS), namely TMEM27 (collectrin) in the kidney proximal tubule and angiotensin converting enzyme 2 (ACE2) in the small intestine [16], [17]. Apart from dependence on organ-specific accessory proteins and recent data on the transcriptional control of its expression along the crypt-villus axis [18], the mechanisms controlling B^0^AT1 expression and function are largely unknown.

Mycoplasma infection represents a well-known and insidious problem for cell culture users. Mycoplasmas are small bacteria (0.3–0.8 µm in diameter) which lack a cell wall and are not susceptible to many usual antibiotics. The major source of mycoplasma contamination in cell culture is the laboratory personnel, as the non-pathogenic mycoplasma (such as *M. orale*) colonize the oral cavity of humans and can easily spread to the laboratory equipment, media and reagents [19]. The effects of mycoplasma infections on the contaminated cell lines are variable and depend on the degree of infection. Previous studies have shown that mycoplasma use arginine as their major source of energy, converting it to ornithine and thereby producing adenosine triphosphate through a complex set of reactions [20]. The lack of an essential amino acid from the cell culture medium induces biochemical changes in the host-cell metabolism, which ultimately lead to a decrease in cell growth and cell death [21].

Since amino acid availability has been shown to affect the expression and activity of several amino acid transporters [22]–[24], the initial goal of the present study was to investigate the effect of extracellular amino acid availability on B^0^AT1 expression, in particular at post-transcriptional levels. To construct an appropriate in vitro proximal tubule model, we co-expressed B^0^AT1 with its renal accessory protein TMEM27 (B^0^AT1-TMEM27) in MDCK cell epithelia under the control of the heterologous cytomegalovirus (CMV) promoter. Unlike for the exogenous expression of antiporters previously mentioned, expression of B^0^AT1-TMEM27 was unstable and inhibited by high extracellular amino acid concentrations. Furthermore, mycoplasma infection of MDCK cells increased expression of all tested exogenously expressed genes presumably via stimulation of the GCN2 pathway.

## Materials and Methods

### Cell culture

MDCK cells, kindly provided by Dr. N. Simmons (Newcastle University) [25] were cultured at 37°C and 5% CO_2_ in DMEM (catalog no. E15-810, GE Healthcare, Glattbrugg, Switzerland) supplemented with 10% heat-inactivated fetal bovine serum (FBS; Sigma-Aldrich, Buchs, Switzerland), 2 mM L-glutamine and 1% non essential amino acids (catalog no. M11-003, GE Healthcare).

Phoenix amphotropic retrovirus producer cells, kindly provided by Dr. G. Nolan (Stanford University) [26], were cultured at 37°C and 5% CO_2_ in DMEM (catalog no. E15-843, GE Healthcare) supplemented with 10% heat-inactivated FBS (Sigma-Aldrich), 2 mM L-glutamine and 1% non essential amino acids.

Human embryonic kidney cells (HEK293), kindly provided by Dr. A. Odermatt (University of Basel) [27] were grown in DMEM (catalog no. E15-810, GE Healthcare) supplemented with 10% heat-inactivated FBS (Sigma-Aldrich), 2 mM L-Glutamine and 1% non essential amino acids at standard cell culture conditions (37°C, 95% relative humidity and 5% CO_2_).

### Mycoplasma detection by PCR analysis and mycoplasma eradication

Mycoplasma infection in cell cultures was detected using Venor GeM (Minerva biolabs, Berlin, Germany) following the manufacturer's instructions. 2 µL of medium deriving from confluent cell cultures were used for each PCR reaction. Mycoplasma infections were eliminated by Ciprofloxacin (Sigma-Aldrich) or Mynox (Minerva biolabs) following the manufacturer's instructions. The eradication was confirmed by PCR analysis.

### Reagents

Physiological medium and arginine-free medium consisted of an amino acid-free DMEM (modified E15-810, custom-made GE Healthcare) supplemented with 5% FBS and physiological amino acid (1× PAA) concentrations or cell culture amino acid (except for arginine) concentrations, respectively, pH adjusted to 7.4, as listed in Table 1. Cell culture tested amino acids, polyamines and urea (Sigma-Aldrich) were dissolved in Hanks' buffered salt solution (HBSS; GE Healthcare) in stock solutions of 10, 50 or 100 mM.

**Table 1:** Amino acid concentrations (µM) in cell culture and physiological media.

Amino Acid	Cell culture medium	Physiological medium
Glycine	500	250
L-Alanine	100	320
L-Arginine Hydrochloride	398	90
L-Asparagine	100	50
L-Aspartic Acid	100	5
L-Cystine Dihydrochloride	201	30
L-Glutamic Acid	100	30
L-Glutamine	5973	660
L-Histidine Hydrochloride-H_2_O	200	90
L-Isoleucine	802	60
L-Leucine	802	120
L-Lysine Hydrochloride	798	200
L-Methionine	201	25
L-Phenylalanine	400	50
L-Proline	100	160
L-Serine	500	110
L-Threonine	798	130
L-Tryptophan	78	90
L-Tyrosine	398	60
L-Valine	803	220

### Cell treatments

Unless specified otherwise, MDCK cells (below passage 10) were seeded at confluent density (1.7·10^5^ cells/cm^2^) and cultivated on Corning Costar Transwell filters (Corning, Amsterdam, The Netherlands) for 6 days in DMEM supplemented with 5% FBS, 2 mM L-glutamine and 1% non essential amino acids. In time course experiments with physiological levels of amino acids, cells were grown in regular DMEM and then treated for the last 1, 3 or 5 days of culture with 1× PAA medium. Cells were grown in regular DMEM for the first 3 days and then treated for 3 additional days with: *1*) amino acid-free medium supplemented with 0.5–8.0× PAA concentrations (dose response experiments); *2*) amino acid-free medium supplemented with the tested amino acids at 8× PAA levels and the remaining amino acids at 0.5× PAA concentrations (substrate specificity experiments). Cells were grown in arginine-free medium supplemented with 180 µM arginine for the first 3 days and then treated for 3 additional days with: *1*) arginine-free medium supplemented with 45, 180 or 720 µM arginine (arginine time course experiments); *2*) arginine-free medium supplemented with 720 µM arginine and NOS inhibitor *N* ^G^-nitro-L-arginine methyl ester (L-NAME, 0–2000 µM; Sigma-Aldrich), arginase inhibitor Nw-Hydroxy-nor-L-arginine (nor-NOHA, 0–500 µM; Millipore, Zug, Switzerland) or α-difluoromethylornithine (DFMO, 0–3 mM; Sigma-Aldrich) (inhibitor experiments); *3*) arginine-free medium supplemented with 45 µM L-arginine in the presence or absence of 675 µM citrulline, ornithine, urea, D-arginine, or 10 µM putresceine, spermidine, spermine, or NO donor sodium nitroprusside (SNP, 0–10 µM; Sigma-Aldrich) (metabolite experiments); *4*) arginine-free medium supplemented with 720 µM arginine and HDAC inhibitor trichostatin A (TSA, 1 µM; Sigma-Aldrich).

### cDNA constructs, transfection, and viral transduction

The human B^0^AT1 cDNA sequence was inserted in the multiple cloning site of pIRES2-EGFP (catalog no. 6029-1, Life Technologies, Zug, Switzerland), upstream of the internal ribosomal entry site and the EGFP reporter gene. Human TMEM27 cDNA sequence was then subcloned in the above mentioned vector in place of EGFP sequence. The resulting bicistronic construct containing B^0^AT1 and TMEM27 upstream and downstream of IRES, respectively, was then excised from the plasmid and inserted in the retroviral vector pLPCX (Clontech, Saint-Germain-en-Laye, France). EGFP was introduced in pLPCX as previously described [28]. Production of supernatants containing the pseudoviruses and subsequent transduction of MDCK target cells was performed as previously described [9]. The first subcultivation after transduction was defined as passage 1. Stable MDCK cell lines were selected and maintained in standard growth DMEM containing 2 µg/mL puromycin (Sigma-Aldrich). Human TMEM27 cDNA sequence was subcloned as a PCR fragment flanked by SmaI and XhoI restriction sites into the Eco47III and XhoI sites of pLenti6-EGFP (Life Technologies), thus yielding to pLenti6-TMEM27 vector. Lentiviral production was performed according to the protocol described elsewhere [29]. Infected MDCK cells were selected with 6 µg/mL blasticidin S (Life Technologies).

### Antibodies

Polyclonal rabbit antibodies were raised against the synthetic peptide NH_2_-NPGLDARIPSLAELEC-CONH_2_ of human B^0^AT1 and further affinity purified (Pineda, Berlin, Germany). Mouse anti-TMEM27 (Abnova, Taipei, Taiwan), mouse anti-EGFP (Clontech), rabbit anti-4E-BP1 (Cell Signaling, Danvers, MA, United States), rabbit anti-phospho-4E-BP1 (Thr70) (Cell Signaling), rabbit anti-eIF2α (Cell Signaling), rabbit anti-phospho-eIF2α (Ser51) (Cell Signaling), rabbit anti-ZO-1 (Life Technologies) and mouse anti-β-actin (Sigma-Aldrich) were used according to the manufacturers' instructions. Horse radish peroxidase goat anti-rabbit IgG and alkaline phosphatase goat anti-mouse IgG secondary antibodies were purchased from Promega (Dübendorf, Switzerland).

### Immunofluorescence staining of MDCK cells

MDCK cells on filters were washed twice with cold phosphate-buffered saline (PBS) supplemented with 1 mM MgCl_2_ and 100 µM CaCl_2_ (PBS^++^) and fixed for 5 min in methanol:acetone (1∶1) at −20°C. Filters were washed three times in PBS^++^ and nonspecific binding sites were blocked for 30 min at room temperature with 2% bovine serum albumin (BSA, Sigma Aldrich) in PBS^++^ supplemented with 0.1% Triton X-100 (Sigma Aldrich). Cells were incubated overnight with primary antibody to anti-B^0^AT1, anti-TMEM27 or anti ZO-1 as indicated, diluted in 2% BSA in PBS^++^ supplemented with 0.1% Triton X-100. After washing, cells were incubated for 1 h at room temperature with Alexa Fluor 594 anti-rabbit-IgG antibody (Life Technologies) and Alexa Fluor 488 anti-mouse IgG antibody (Life Technologies). 4′,6-Diamidino-2-Phenylindole Dihydrochloride (DAPI, Life Technologies) was added to the secondary antibody mix in order to counterstain the nuclei. Filter pieces were mounted in DAKO-Glycergel (Dako, Baar, Switzerland) and analyzed with a Leica TCS SP5 confocal laser scanning microscope (Leica, Heerbrugg, Switzerland) using a 63× objective lens (Leica). Digital images were processed using the software Imaris (Bitplane, Zurich, Switzerland).

### Amino acid uptake

MDCK cells were grown on filters and the trans-epithelial electrical resistance across intact monolayers was measured using EVOHM device (World Precision Instruments, Sarasota, FL). Amino acid uptake was performed as previously described [9]. Briefly, cells were washed three times and then incubated for 30 min at 37°C with uptake buffer (150 mM NaCl, 10 mM HEPES pH 7.4, 1 mM CaCl_2_, 5 mM KCl, 1 mM MgCl_2_, 10 mM glucose). Fresh uptake buffer was then applied on the basolateral side whereas the apical compartment received the uptake buffer supplemented with 1 mM L-Leucine and the corresponding ^3^H-labeled L-leucine (Hartmann Analytic, Braunschweig, Germany) as tracer. ^14^C-labeled mannitol (Hartmann Analytic) was used as a control for the integrity of the cell monolayer. After 10 min incubation at 37°C, the uptake was stopped by replacing the apical and basolateral solutions with ice-cold uptake buffer. The cells were washed three times and the filters were excised and placed into scintillation fluid and shaked overnight at room temperature. Radioactivity was measured by liquid scintillation analyzer (Packard Tri-Carb 2900TR, PerkinElmer, Schwerzenbach, Switzerland).

### Western blotting

Cells were lysed with ice-cold Radio-Immunoprecipitation Assay (RIPA) buffer (150 mM NaCl, 50 mM Tris pH 7.4, 1% NP-40, 0.5% Na-Deoxycholate) supplemented with fresh Protease Inhibitor Cocktail (Sigma) and PhosSTOP (Roche, Rotkreuz, Switzerland) and incubated for 40 min on ice. Cellular debris was pelleted with centrifugation at 2500 g for 10 min at 4°C and the protein concentration was determined by DC-Protein Assay, following the manufacturer's guidelines (Bio-Rad, Cressier, Switzerland). Cell extracts (20–40 µg protein) were resolved by SDS-PAGE on 10 or 12% gels and electrophoretically transferred to PVDF membranes (Immobilion-P, Millipore). Nonspecific binding sites were blocked for 1 h at room temperature with 5% powdered milk in Tris-buffered saline supplemented with 0.1% Tween-20 (TBS-T). Blots were then incubated overnight at 4°C with the primary antibody diluted in 5% powdered milk in TBS-T. After washing the blots with TBS-T, secondary antibody diluted in 5% powdered milk in TBS-T was applied for 1 h at room temperature and the antibody binding was detected with Immobilion Western Chemiluminescent HRP substrate (Millipore) or CDP-Star (Roche) and visualized with FujiFilm Las-4000 camera (GE-Healthcare) according to the manufacturer's instructions. Image-J software (National Institutes of Health, Bethesda, MD) was used for densitometric analysis of the Western blots.

### RT-PCR

Total RNA from cell pellet was isolated using RNeasy Mini Kit (Qiagen, Basel, Switzerland) following the manufacturer's instructions. Reverse transcription of the isolated RNA was performed with TaqMan RT Kit using random hexamers (Life Technologies). Gene expression was quantified by quantitative real-time PCR using Taq Ready Mix (Life Technologies) as previously described [12]. Primers and probes are listed in Table 2. Probes were labelled with the reporter dye FAM at the 5′ end and the quencher dye TAMRA at the 3′ end (Microsynth, Balgach, Switzerland). β-Actin or 18S (Life Technologies) were used as housekeeping genes.

**Table 2:** List of primers and probes used for quantitative real-time PCR.

Target mRNA	Accession number	Sequence (5′→3′)
β-Actin	NM_001195845.1	Sense: CAAGGTTGGGGACTTAGCTG
		Antisense: GAGTAGAGTGAGCATGAGATCCAG
		Probe: #97 (Cat no. 04692144001, Roche)
B^0^AT1	NM_001003841.2	Sense: GTGTGGACAGGTTCAATAAGGACAT
		Antisense: CCACGTGACTTGCCAGAAGAT
		Probe: TCATGATCGGCCACAAGCCCAA
TMEM27	NM_020665.5	Sense: CCTCTTCAAAGCGATGGTAGCT
		Antisense: CCCTCTGGGTTACATTGCAAA
		Probe: CCCAACAGAGAAGCAACAGAAATTTCCCA
eGFP	YP_002302326.1	Sense: GTCCGCCCTGAGCAAAGA
		Antisense: TCCAGCAGGACCATGTGATC
		Probe: CCCAACGAGAAGCG

### Amino acid measurements

Media were collected from MDCK cells grown on filters. Briefly, deproteinized samples were derivatized using EZ:faast kit (Phenomenex, Torrance, CA) and further analyzed by LC-MS/MS using API Sciex 2000 instrument (Ab Sciex, Brugg, Switzerland).

### Cell count

Cell nuclei were visualized using 4′,6-Diamidino-2-Phenylindole Dihydrochloride (DAPI, Life Technologies) and quantified using Image J software (National Institute of Health, USA). Briefly, the color image (RGB) was converted to grayscale (8-bit) and the threshold was adjusted. Watershed function was used to divide particles which had merged together. Nuclei were then counted, setting the size (pizel∧2) between 400 and 3000 and circularity as default (0.00–1.00). The number of counted particles was then corrected for the area of the filter using Microsoft Excel Software.

### Statistical analysis

All experiments were carried out in at least 3 independent replicates. Data are expressed as mean ± SEM. Analysis of the experimental data was performed by GraphPad Prism 5.0.

## Results

### Co-expressed exogenous B^0^AT1 and TMEM27 localize to the luminal membrane of MDCK cells

**Figure 1:**
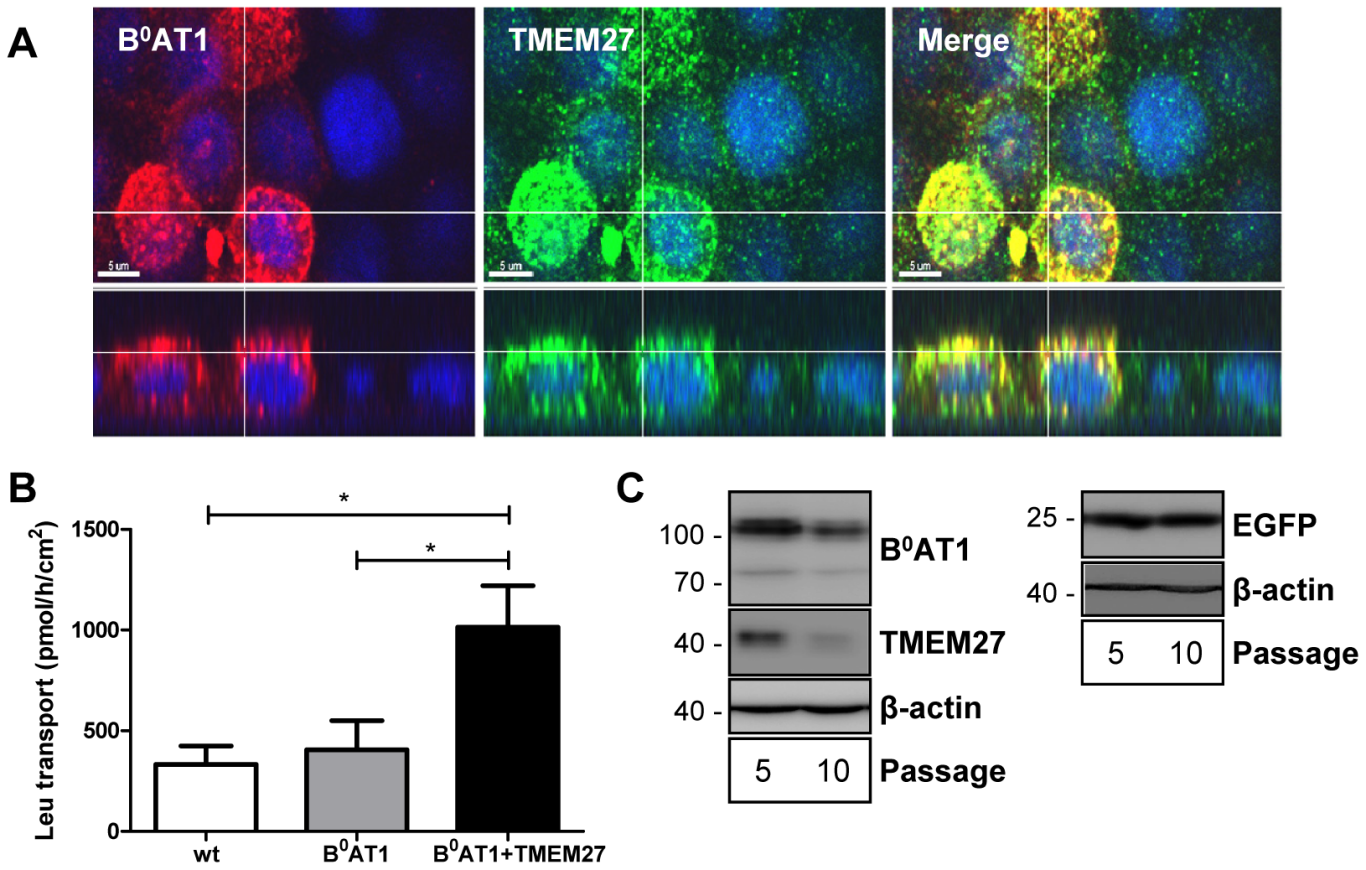
Overexpression of B^0^AT1 and TMEM27 in MDCK cells. A: immunofluorescence analysis of B^0^AT1-TMEM27 overexpressing MDCK cells. Apical co-localization (yellow) of B^0^AT1 (red) and TMEM27 (green) is visualized by confocal microscopy. Upper panels represent images taken parallel to the filter (x–y plane); lower panels show corresponding z–x reconstitutions. Bars  = 5 µm. B: Apical uptake of L-Leu (100 µM, 10 min) in wild type (wt) or B^0^AT1 or B^0^AT1-TMEM27 overexpressing MDCK cells cultivated on filters. Data are represented as mean ± SEM (n = 3). Groups were compared by one-way ANOVA followed by Tukey post-test; * p≤0.05. C: MDCK cells overexpressing B^0^AT1-TMEM27 or EGFP. After viral transduction, cells were subcultured on plastic dishes for 5 or 10 times (passage 5 and 10, respectively) in standard cell culture medium. Western blotting experiments with antibodies directed against B^0^AT1, TMEM27 and EGFP were performed. Representative Western blotting images of 3 independent experiments are shown.

To investigate the impact of amino acids on the expression of the major renal proximal tubule luminal sodium-neutral amino acid symporter B^0^AT1-TMEM27, we established MDCK cells overexpressing both transporter subunits using a retroviral system. Such an *in vitro* system was successfully employed for previous studies on overexpressed luminal amino acid antiporter b^0,+^AT-rBAT (SLC7A9-SLC3A1) and basolateral y^+^LAT1-4F2hc (SLC7A7-SLC3A2) and LAT2-4F2hc (SLC7A8-SLC3A2) [8], [9]. Immunofluorescence studies on polarized MDCK cells showed an apical co-localization of B^0^AT1 and TMEM27 when co-expressed (Fig. 1A). Consistent with previous observations in other expression systems [16], TMEM27 co-expression increases B^0^AT1 transport function also at the luminal surface of cultured MDCK cell epithelia (Fig. 1B).

### Expression of exogenous B^0^AT1 and TMEM27 is prevented by high amino acid content of cell culture medium and regulated upon mycoplasma infection

**Figure 2:**
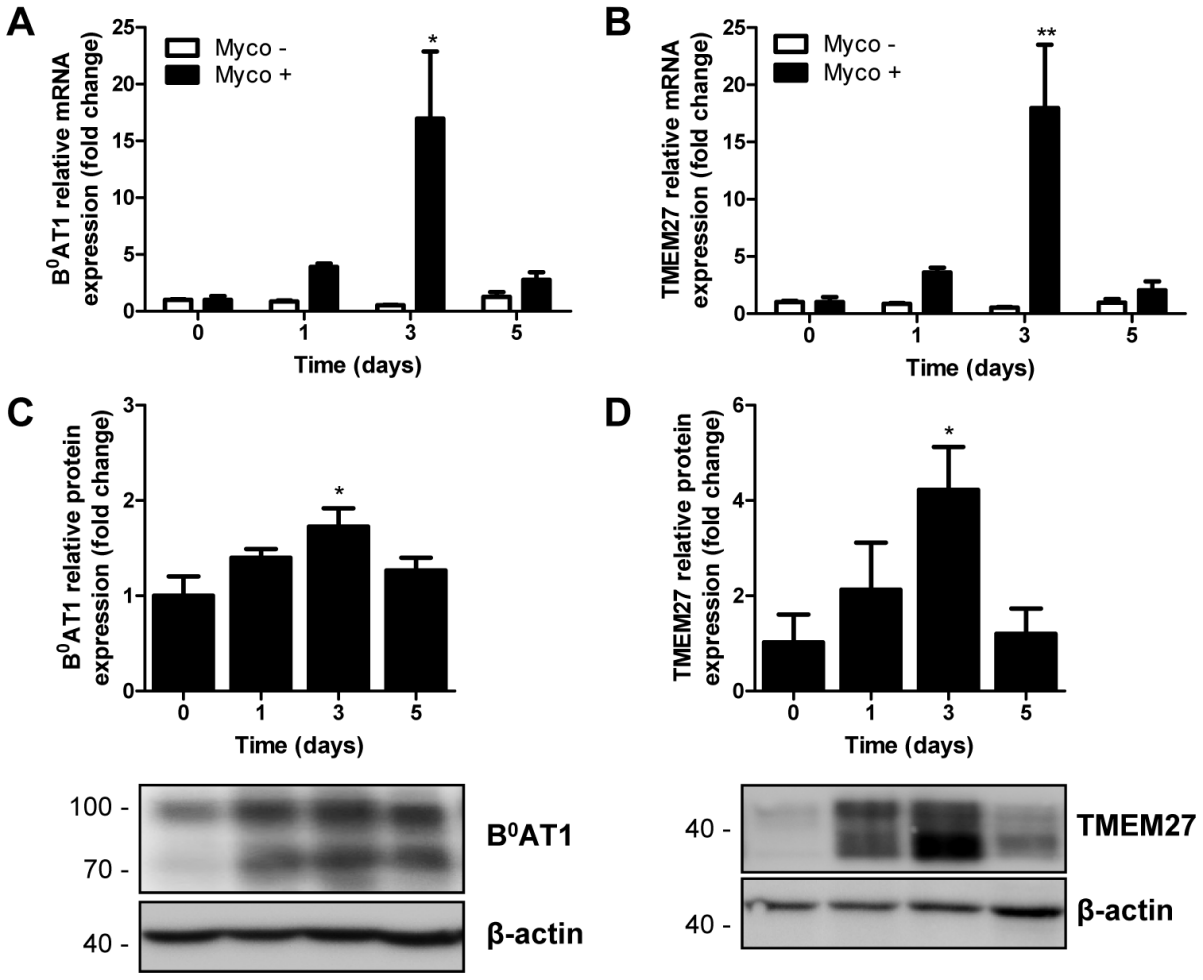
Effect of physiological amino acid levels on transgenes expression. Cells were cultivated on filters in standard cell culture medium and treated for the indicated times with physiological medium. A–B: mRNA expression of B^0^AT1 (A) or TMEM27 (B) was measured by quantitative PCR-analysis in B^0^AT1-TMEM27 overexpressing MDCK cells which were found mycoplasma-free (open bar) or mycoplasma-infected (black bar). mRNA levels were standardized to β-actin and normalized to time 0. C–D: Western blotting experiments with antibodies directed against B^0^AT1 (C) or TMEM27 (D) were performed in mycoplasma-infected B^0^AT1-TMEM27 overexpressing MDCK cells and the intensity of the immunoreactive bands was quantified, standardized to β-actin and normalized to time 0. Representative Western blotting images are shown. Data are represented as mean ± SEM (n = 3). Groups were compared by one-way ANOVA followed by Dunnett post-test; ** p≤0.01, * p≤0.05.

As previously observed in various attempts to (over)express B^0^AT1 and TMEM27 in MDCK cells (data not shown), the expression of B^0^AT1 and TMEM27 proteins strongly decreased following subsequent cell culture passages (Fig. 1C and 1D). This effect was specific for the amino acid transporter and its accessory protein as the control cell line overexpressing EGFP did show a less significant change in protein expression. We speculated that in the elevated extracellular amino acid concentrations in standard cell culture medium the concentrative transporter B^0^AT1 increases intracellular amino acids concentrations. Increased intracellular levels exert a negative feedback on the activity and/or expression of the transporter, resulting in its downregulation. To test this hypothesis, freshly transduced B^0^AT1-TMEM27 overexpressing MDCK cell lines were subcultured on plastic dishes in low amino acids containing media (so called physiological medium) or in control media (classical DMEM) and transgene protein expression was assessed by Western blotting. Unexpectedly, physiological amino acid containing medium did not significantly increase B^0^AT1-TMEM27 expression after 10 passages (Fig. S1). To test the effect of amino acid concentrations on polarized epithelia, B^0^AT1-TMEM27 overexpressing MDCK cell lines were cultivated on filters in physiological or standard cell culture medium and transgene mRNA and protein expression was assessed by quantitative PCR and Western blotting, respectively. When comparing various B^0^AT1-TMEM27 overexpressing MDCK cell lines under otherwise identical experimental conditions, we observed considerable variations in the response to physiological medium. PCR-based test for the presence of mycoplasma revealed an infection of these cell lines (data not shown) explaining their observed phenotypic change. Interestingly, B^0^AT1-TMEM27 overexpressing mycoplasma-infected MDCK cells cultured in low amino acid media reproducibly demonstrated a rapid time-dependent increase of transgene mRNA (Fig. 2A and 2B) and protein expression (Fig. 2C and 2D), with a peak at three days. The effect was specific for mycoplasma-infected cells as neither mycoplasma-free cells (Fig. 2A and 2B, open bar) nor cells which were infected by mycoplasma and then treated with antibiotics (data not shown) showed a regulation of transgene expression. However, this effect of culture in low amino acid media was not specific for B^0^AT1-TMEM27 expression. Indeed, mycoplasma-infected MDCK cell lines overexpressing EGFP or TMEM27 in the absence of B^0^AT1 displayed a similar upregulation of transgene mRNA expression in low amino acid medium (Fig. S2). In contrast, the mRNA expression of endogenous housekeeping genes such as GAPDH and β-actin was unaffected (data not shown). Taken together, these results suggested that in low amino acid medium mycoplasma infection upregulates mRNA and protein expression of transgenes in MDCK cells.

### Mycoplasma-mediated L-arginine depletion stimulates exogenous B^0^AT1 and TMEM27 expression

**Figure 3:**
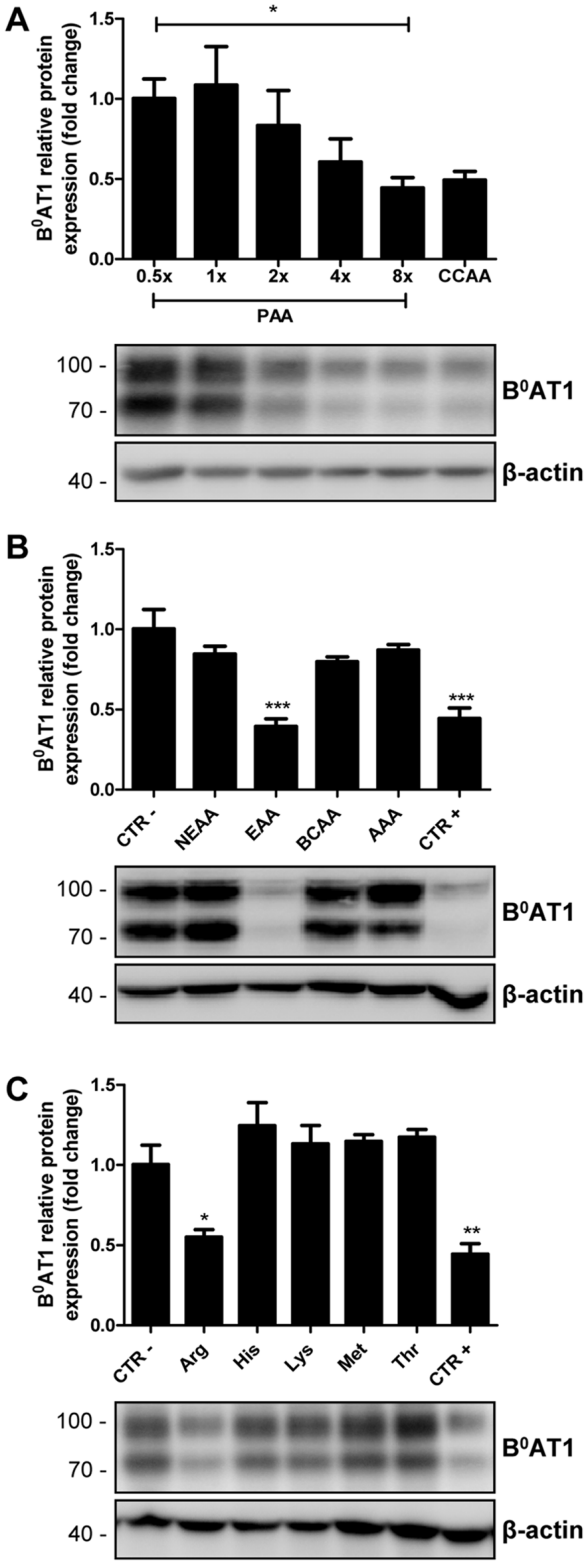
Transgene regulation by amino acids. A: Mycoplasma-infected B^0^AT1-TMEM27 overexpressing MDCK cells were incubated in standard cell culture medium (CCAA) or in amino acid-free medium complemented with different amounts (0.5× to 8×) of an amino acid mixture corresponding to the concentrations found in post-absorptive mouse plasma (PAA doses). B^0^AT1 expression was analyzed by Western blotting and the intensity of the immunoreactive bands was quantified, standardized to β-actin and normalized to the level measured in 0.5 fold complemented medium. B-C: Mycoplasma-infected B^0^AT1-TMEM27 overexpressing MDCK cells were incubated in a modified medium containing the indicated groups (B) or single (C) amino acids (NEAA: non essential amino acids – Ala, Asn, Asp, Cys, Glu, Gln, Gly, Pro and Ser; EAA: essential amino acids – Arg, His, Lys, Met and Thr; BCAA: branched chain amino acids – Ile, Leu and Val; AAA: aromatic amino acids – Phe, Trp and Tyr) at 8× their normal plasma concentrations whereas the remaining amino acids were given at 0.5× their normal plasma level. CTR − and + represent cells which were treated with all the amino acids at 0.5× and 8.0× their plasma level, respectively. B^0^AT1 expression was analyzed by Western blotting and intensity of the immunoreactive bands was quantified, standardized to β-actin and normalized to CTR −. Representative Western blotting images are shown. Data are represented as mean ± SEM (n = 3). Groups were compared by one-way ANOVA followed by post-test for linear trend (A) or Dunnett post-test (B–C); *** p≤0.001, ** p≤0.01, * p≤0.05.

Next, we evaluated the dose response effect of media amino acids on transgene expression in mycoplasma-infected B^0^AT1-TMEM27 overexpressing MDCK cells. Western blotting analysis of protein samples prepared from cells cultured in different amino acid concentrations revealed a dose-dependent effect on B^0^AT1 expression (Fig. 3A).

To identify whether a specific amino acid was responsible for the observed transgene upregulation, we divided the 20 proteinogenic amino acids into four groups and tested their effect on B^0^AT1 expression. Western blotting experiments showed that elevated essential amino acids alone were able to reduce B^0^AT1 protein expression as strongly as the control media (8-fold plasma amino acid concentrations which is similar to standard culture medium). In contrast, none of the other amino acid groups affected B^0^AT1 protein abundance (Fig. 3B). Therefore, the five essential amino acids of the group were tested individually. Only arginine, of the tested amino acids, significantly regulated B^0^AT1 protein abundance (Fig. 3C). These data showed that in mycoplasma-infected MDCK epithelia transgene upregulation by low amino acid media is dose dependent and suggested a pivotal role for arginine.

**Figure 4:**
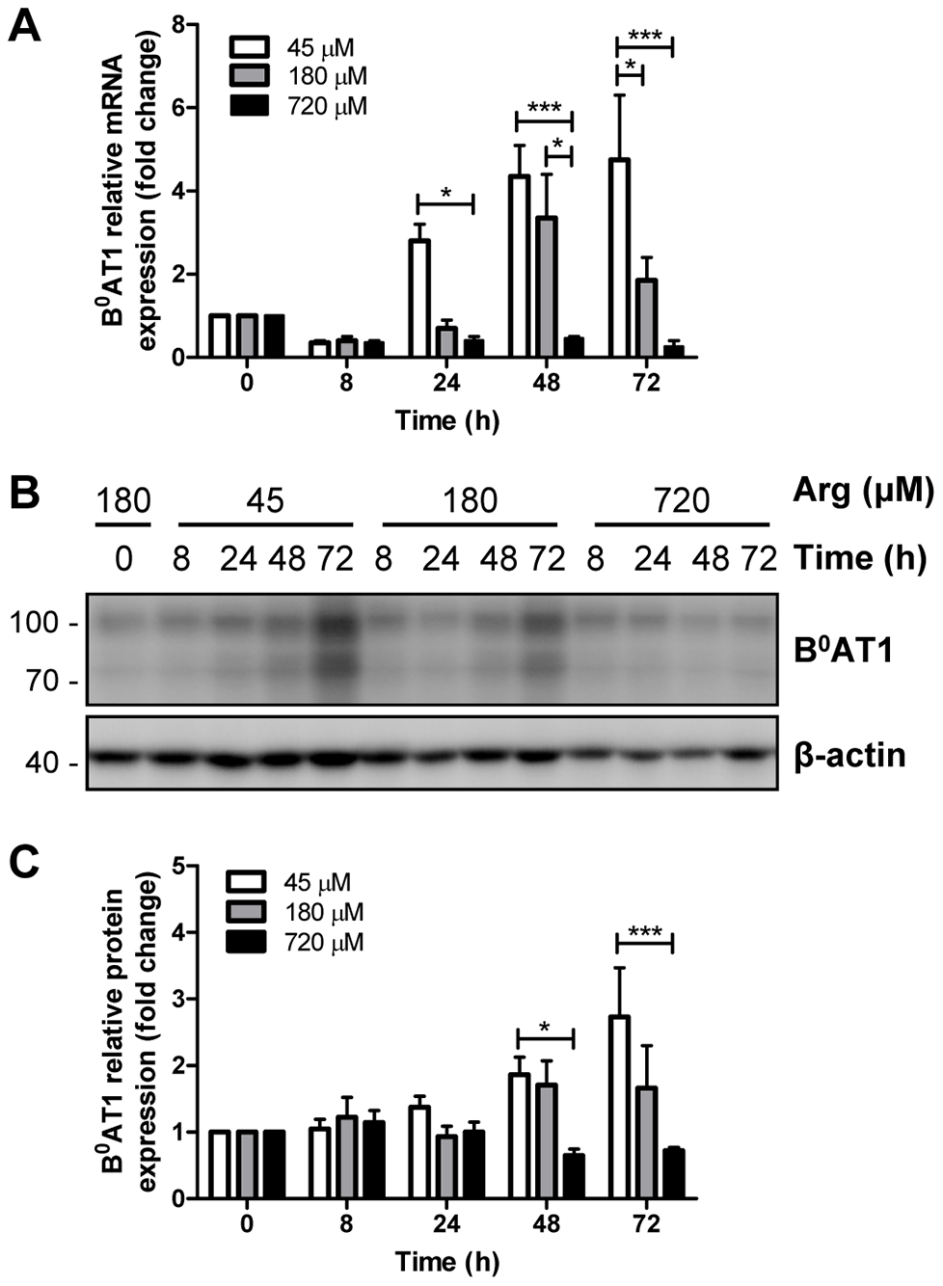
Time course and concentration dependence of effect of arginine on transgene expression. Mycoplasma-infected B^0^AT1-TMEM27 overexpressing MDCK cells were cultivated in arginine-free DMEM supplemented with 45, 180 or 720 µM arginine for the indicated times. A: Real-time PCR quantification of B^0^AT1 mRNA expression. mRNA levels were standardized to β-actin and normalized to time 0. B: Representative Western blotting of B^0^AT1 expression. C: the intensity of B^0^AT1 immunoreactive bands was quantified, standardized to β-actin and normalized to 0 h. Data are represented as mean ± SEM (n = 3). Groups were compared by two-way ANOVA followed by Bonferroni post-test; *** p≤0.001, * p≤0.05.

To investigate the specific role of arginine on transgene expression, time course and dose response experiments were performed (Fig. 4). Quantitative PCR analysis showed that low arginine concentration drastically increases B^0^AT1 mRNA expression in a time-dependent manner (Fig. 4A), whereas transgene mRNA expression was inhibited by high arginine concentrations. Likewise but to a lesser extent, B^0^AT1 protein abundance was also regulated by low arginine concentrations in a time-dependent manner (Fig. 4B and 4C). Thus, these data indicated that in mycoplasma-infected MDCK cells low arginine levels increase both transgene mRNA and protein expression.

### Transgene expression is not regulated by mammalian arginine metabolism

**Figure 5:**
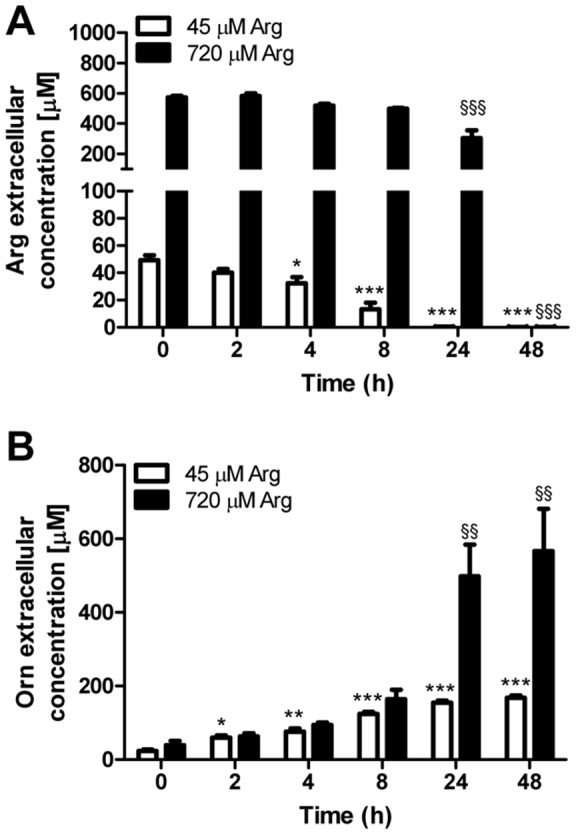
Time course of arginine metabolism in low and high arginine medium. Concentrations of arginine (A) and ornithine (B) were measured in the medium of mycoplasma-infected B^0^AT1-TMEM27 overexpressing MDCK cells treated with 45 µM (open bar) or 720 µM (black bar) arginine at the indicated times. Groups were compared by one-way ANOVA followed by Dunnett post-test; *** p≤0.001, ** p≤0.01, * p≤0.05 versus 45 µM arginine; ^§§§^ p≤0.001, ^§§^ p≤0.01, versus 720 µM arginine.

The arginine effect on transgene expression appeared to have a relatively slow time course, suggesting the possibility that amino acid metabolism plays a critical role. We analyzed the amino acid concentration in the media of mycoplasma-infected MDCK cells cultured in low and high arginine media. Our data showed that arginine was indeed depleted from the medium within 24 h when initially administered at low concentration (Fig. 5A). Even when given at high concentration arginine metabolism led to its depletion within 48 h. This increased amino acid metabolism was arginine-specific as all other amino acids were not significantly decreased in the medium after 48 h (data not shown). Arginine consumption rate was correlated with ornithine production (Fig. 5B). Citrulline was also measured but its concentration was below the detection limits (<10 µM) at all the time points, except for the 48 h (7.1±3.9 µM in low arginine medium and 13.1±1.7 µM in high arginine medium).

**Figure 6:**
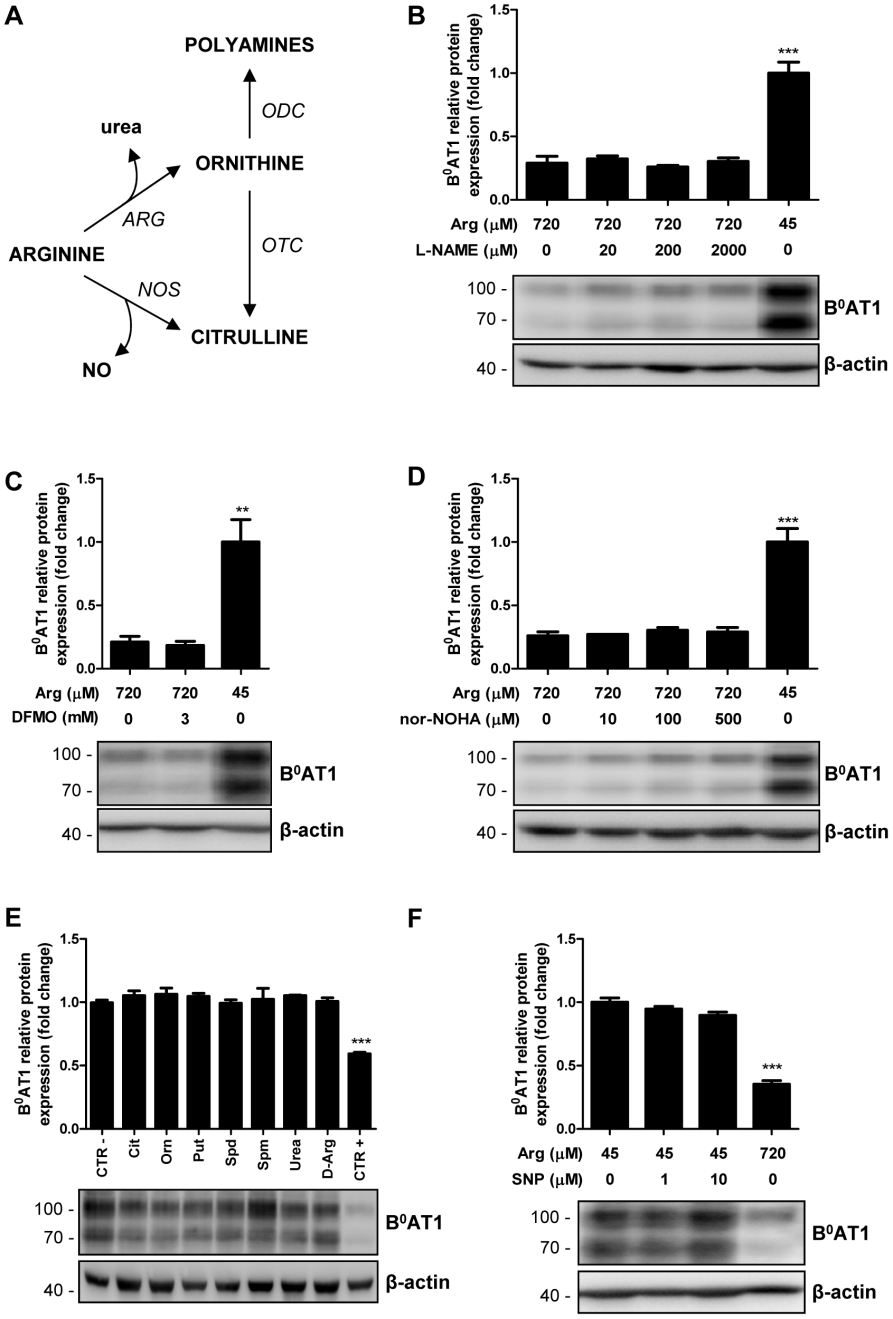
Effect of L-arginine metabolism on transgene expression. A: Schematic overview of some mammalian arginine metabolic pathways. ARG: arginase; NO: nitric oxide; NOS: nitric oxide synthase; ODC: ornithine decarboxylase; OTC: ornithine transcarbamylase. B–D: Mycoplasma-infected B^0^AT1-TMEM27 overexpressing MDCK cells were cultivated in arginine-free DMEM supplemented with 45 or 720 µM arginine in the presence or absence of the indicated concentrations of inhibitors of the following enzymes: (B) nitric oxide synthase (N^G^-nitro-L-arginine methyl ester, L-NAME); (C) ornithine decarboxylase (α-difluoromethylornithine, DFMO) and (D) arginase (Nw-Hydroxy-nor-L-arginine, nor-NOHA). E–F: Mycoplasma-infected B^0^AT1-TMEM27 overexpressing MDCK cells were cultivated in arginine-free DMEM supplemented with 45 µM arginine in the absence (negative control, CTR −) or presence of (E) the indicated metabolites or (F) a nitric oxide donor (sodium nitroprusside, SNP). Positive control (CTR +) represents cells treated with 720 µM arginine. B^0^AT1 expression was analyzed by Western blotting and intensity of the immunoreactive bands was quantified, standardized to β-actin and normalized to 45 µM arginine. Representative Western blotting images are shown. Data are represented as mean ± SEM (n = 3). Groups were compared by one-way ANOVA followed by Dunnett post-test; *** p≤0.001 ** p≤0.01.

Next, we tested whether inhibitors of mammalian arginine metabolizing enzymes, in particular of arginase, nitric oxide synthase and ornithine decarboxylase impact on B^0^AT1 expression (see Fig. 6A for pathways). The fact that none of the tested drugs had an effect on B^0^AT1 protein expression suggested that their products are not involved in the observed transgene regulation (Fig. 6 B–D). Consistent with this observation, incubation of the transduced MDCK cells with high doses of ornithine, urea, polyamines, citrulline or a NO donor also did not affect transgene expression (Fig. 6E and 6F). Furthermore, the effect on transgene expression seemed to be specific for L-arginine, as D-arginine did not exert any effect (Fig. 6E). Thus, these results suggested that the observed transgene upregulation was not mediated by products of L-arginine metabolism but by the lack of L-arginine.

### GCN2 pathway is activated by low arginine levels in mycoplasma-infected MDCK cells

**Figure 7:**
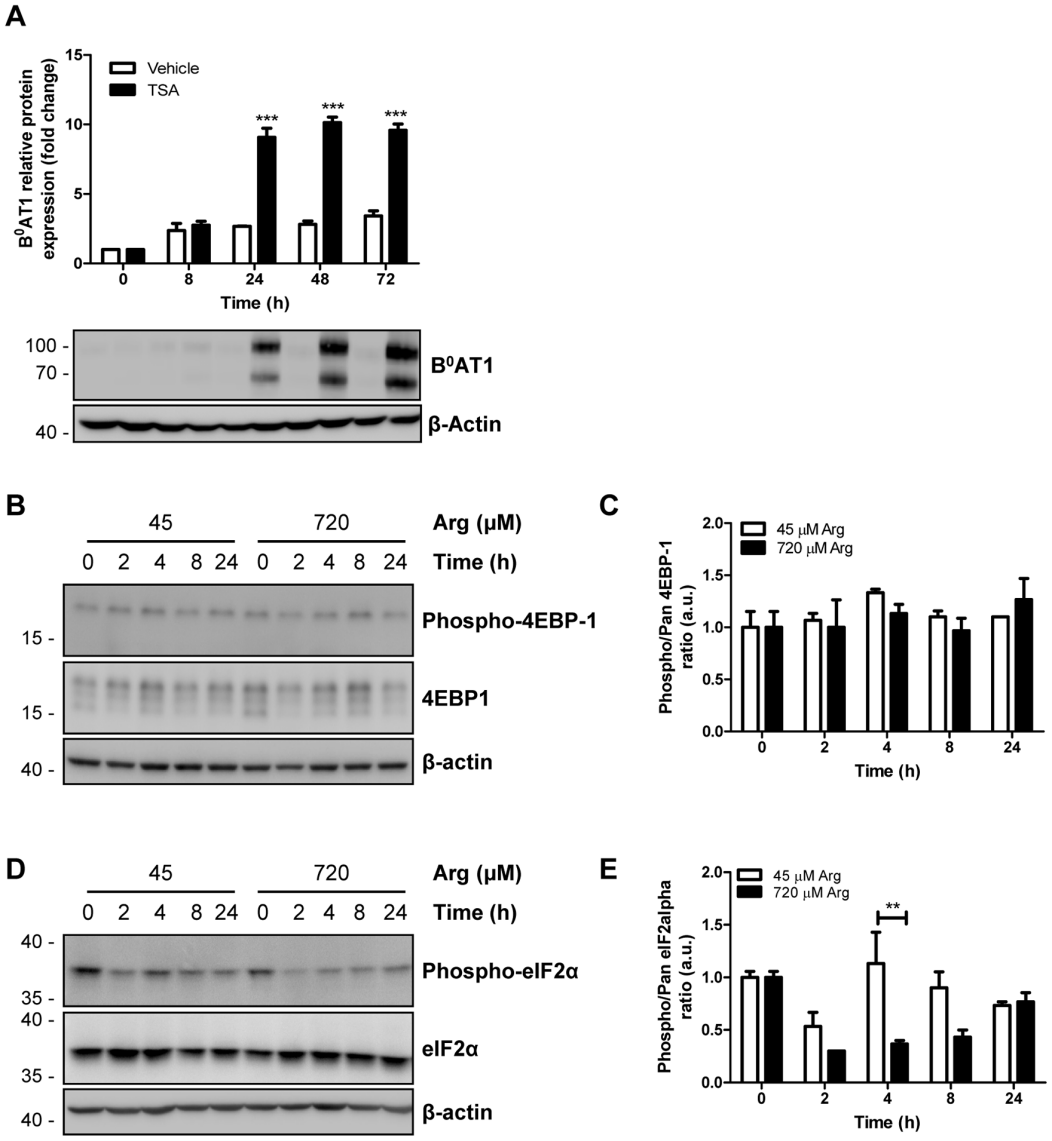
Effect of arginine on cell signaling. A: Mycoplasma-infected B^0^AT1-TMEM27 overexpressing MDCK cells were grown in standard cell culture medium supplemented with an inhibitor of histone deacetylation (TSA, 1 µM) for the indicated times. B^0^AT1 expression was analyzed by Western blotting and intensity of the immunoreactive bands was quantified, standardized to β-actin and normalized to time 0. Representative Western blotting images are shown. Data are represented as mean ± SEM (n = 3). Groups were compared by two-way ANOVA followed by Bonferroni post-test; *** p≤0.001. B–E: Mycoplasma-infected B^0^AT1-TMEM27 overexpressing MDCK cells were incubated with 45 or 720 µM arginine medium for the indicated times and the whole cell protein extracts were subjected to immunoblotting for total- and phospho-4EBP-1 (B–C) and eIF2α (D–E). Representative Western blotting images are shown in B and D. The degree of 4EBP-1 (C) and eIF2α (E) phosphorylation was assessed by quantifying the immunoreactive bands of the phosphorylated form and normalizing to the total protein in each lane. Data are represented as mean ± SEM (n = 3). Groups were compared by one-way ANOVA followed by Dunnett post-test; ** p≤0.01.

Recent studies have shown that transgene expression is regulated by low levels of essential amino acid such as Tyr and Met/Cys which trigger a downregulation of the histone deacetylase 4 (HDAC4) [30]. Indeed, inhibition of HDAC activity by trichostatin A (TSA) also upregulated the protein expression of B^0^AT1 (Fig. 7A). However, the mRNA expression of HDAC 4 as well as that of others HDACs (2, 3, 5, 6, 8, 10 and 11) was not regulated by a decrease in arginine concentration (data not shown).

**Figure 8:**
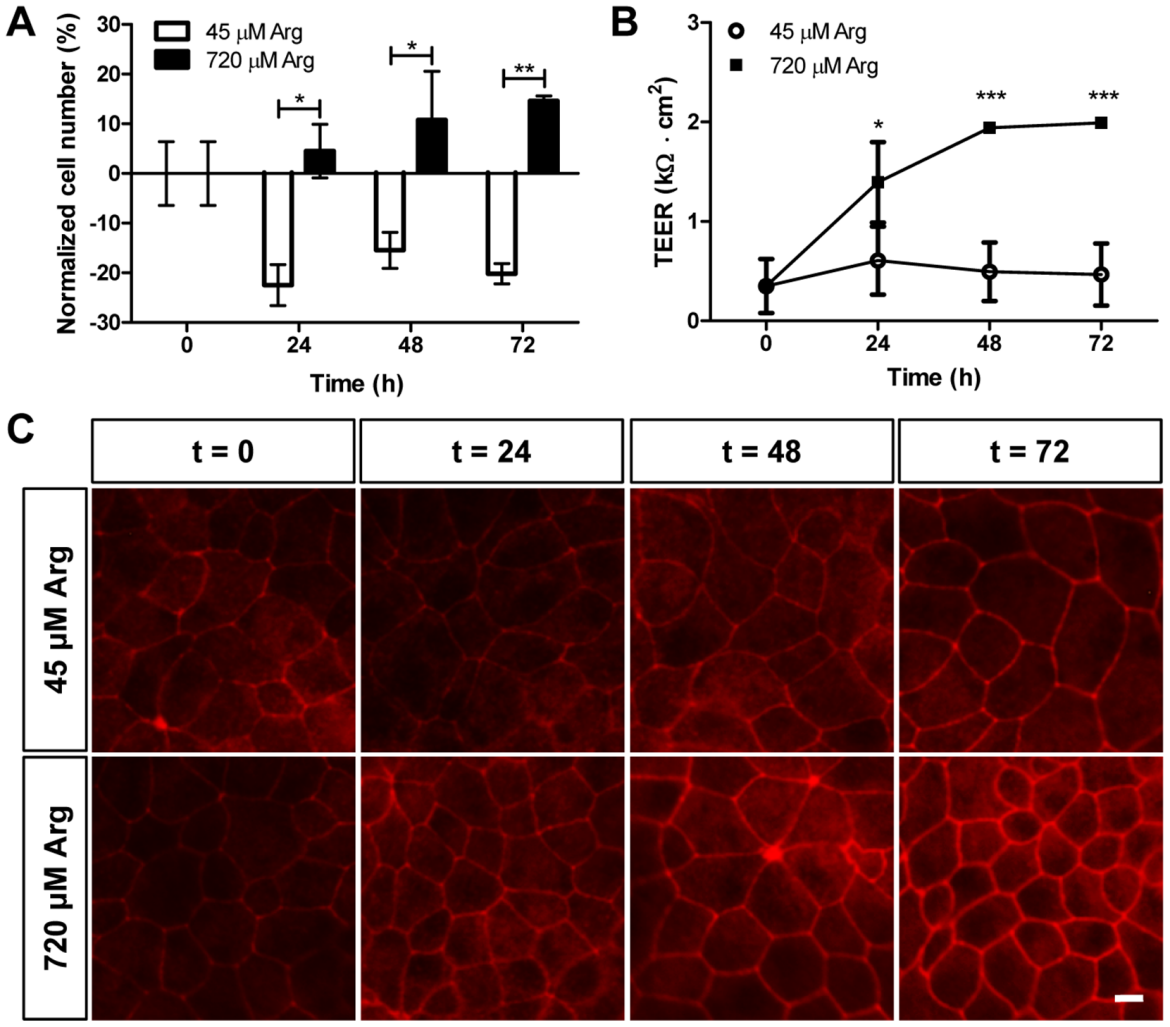
Effect of arginine on cell number and tight epithelium. Mycoplasma-infected MDCK wild type (wt) cells were cultivated in arginine-free DMEM supplemented with 45 or 720 µM arginine for the indicated times. A: Cell number was estimated based on DAPI-staining followed by Image J analysis as described in Materials and Methods. B: Trans-epithelial electrical resistance (TEER) was measured every 24 h using EVOHM device. Data in panels A and B are represented as mean ± SEM (n = 3). Groups were compared by two-way ANOVA followed by Bonferroni post-test; *** p≤0.001, ** p≤0.01, *  = p≤0.05. C: Immunofluorescence analysis of representative wt MDCK cells with antibodies raised against the tight junction protein ZO-1. Scale bar is 10 µm and applies to all panels.

AA availability is known to be sensed by mTOR and the GCN2 pathway. To investigate whether mycoplasma-induced arginine depletion results in the regulation of one of these two pathways, the phosphorylation of their downstream effectors 4EBP-1 and eIF2α, respectively, was measured by Western blotting. While the phosphorylation of the mTOR downstream effector 4EBP-1 was unaffected by low arginine levels (Fig. 8A and 8B), the phosphorylation of the GCN2 effector eIF2α was rapidly increased after 4 h of incubation in low arginine medium (Fig. 8C and 8D). Interestingly, in the presence of high arginine medium eIF2α phosphorylation was also increased, but with a much slower time course. Taken together, these data showed that mycoplasma infection of MDCK cell cultures causes a rapid L-arginine depletion, which in turn activates the GCN2 pathway.

### Mycoplasma-induced arginine depletion affects MDCK cell number and tight epithelium formation

The GCN2 pathway activation leads to a global decrease in protein synthesis, which in turn affects several vital processes such as proliferation, differentiation and apoptosis [5]. Indeed, mycoplasma-infected MDCK wild type cells showed a 20% reduction in cell number after 24 h culture in low arginine medium (Fig. 8A). In contrast, cells cultured in high arginine medium showed a progressive increase in cell number within 72 h. We also found that mycoplasma-infected MDCK cells cultivated on porous filters with low arginine medium presented low trans-epithelial electrical resistance (TEER) during the time of culture (Fig. 8B). In contrast, MDCK cells cultured in high arginine medium showed an increase in TEER over time, compatible with the formation of epithelial tight junctions. To determine if the effect of arginine on trans-epithelial electrical resistance was due to the regulation of tight junction proteins expression, we performed immunofluorescence staining of ZO-1, a classical component of tight junctions [31]. Our data clearly showed that mycoplasma-infected MDCK cells initially had a low expression of ZO-1 at the tight junctions when treated with low arginine medium (Fig. 8C, t = 24 h). Nevertheless, both arginine treatments resulted in comparable ZO-1 protein abundance at the end of the cell culture (Fig. 8C, t = 72 h), suggesting that low arginine medium retards but does not fully prevent tight junction formation. These results showed that arginine deprivation dramatically affects MDCK epithelia, resulting in a decrease in cell number and TEER, which in turn corresponds to a delay in tight junction formation.

## Discussion

The aim of this study was to characterize the impact of amino acids on the expression of the neutral amino acid transporter B^0^AT1 and its accessory protein TMEM27 in MDCK cells. The rationale for this study was based on the observation that B^0^AT1-TMEM27 overexpressing MDCK cells displayed progressively reduced transgene expression after only a few culture passages. Interestingly, infection of MDCK cells with mycoplasma led to a rapid arginine depletion, which in turn triggered the derepression of silenced transgenes, most likely through the activation of the GCN2 pathway. These findings expand the biochemical changes induced by mycoplasma to the metabolism of infected cells by adding the novel role for arginine in the control of transgene expression [21]. Transgene silencing represents a common defense mechanism of mammalian cells, which identify the foreign sequences as cellular invaders and target them for silencing. Recent studies have shown that limitation of essential amino acids such as Tyr or Met/Cys regulates transgene expression in mammalian cells [30]. Here, we show that another (conditionally) essential amino acid, namely arginine can affect transgene expression. However, these results are in variance with those of Palmisano et al. [30], who suggested that the transgene regulation is a process involving inactivation or downregulation of the histone deacetylase 4 (HDAC4). Although both studies agree that amino acid deprivation activates a general response able to derepress integrated transgenes, we could not confirm the regulation of the transgene by histone deacetylases. Instead, the decrease of arginine in the medium was correlated with an increase in eIF2α phosphorylation after 4 h, which suggests that the transgene regulation is mediated by the activation of the GCN2 pathway. The amino acid response induced by amino acid deprivation is known to regulate gene expression at many steps, from the chromatin structure to the transcription and translation rates [1], [32]. However, the mechanism which controls chromatin structure remodeling in response to amino acid deprivation is not yet understood [1]. Interestingly, the expression of several genes encoding transcription factors has been shown to be increased by amino acid limitation. Examples include members of the activating transcription factor (ATF) family, FOS/JUN family, CCAAT/enhancer binding protein (C/EBP) family, and other transcription factors outside of the bZIP superfamily (reviewed in Ref. [33]). To date, there are no known transcription factors that increase the CMV promoter transcription rate resulting in an increase of the mRNA expression of transgenes. Instead, luciferase constructs driven by CMV promoter have been shown to be unaffected by amino acid deprivation in skeletal muscle cells [34].

In the present study, inhibition of mammalian arginase, nitric oxide synthase and ornithine decarboxylase did not affect the transgene expression in MDCK cells. The rapid and massive breakdown of arginine to ornithine in MDCK cells is consistent with the time course previously observed in HeLa cells infected with mycoplasma [20]. Furthermore, arginine deiminase, the first mycoplasma enzyme involved in arginine breakdown, has a much higher arginine affinity (Km ∼30 µM) than mammalian arginases (Km ∼45 mM) [35]. Thus, although arginase activity of the cell line could account for some ornithine production, it is unlikely that the observed arginine degradation is an intrinsic feature of MDCK cells, but more likely it is an effect of mycoplasma infection. Given that the reaction which converts arginine to ornithine has a stoichiometry of 1, it was surprising to observe that ornithine concentration in the low arginine medium exceeded by far the expected concentration. It is possible that part of measured ornithine derives from the metabolism of glutamine and glutamate, which are present at approximately 6 mM in the cell culture medium.

In addition to the effect on transgene expression, we found that mycoplasma-induced arginine depletion has an overall impact on MDCK epithelium formation. The reduction in MDCK cell number is consistent with previous studies which showed that deprivation of essential amino acids from the cell culture medium (including arginine) triggers cell death [36], [37]. Interestingly, cells treated with high arginine concentrations increased in cell number. However, the phosphorylation of 4E-BP1 protein was not affected by arginine levels in the media, suggesting that the mTOR pathway does not respond to arginine under the tested conditions in MDCK cells. These findings differ from previous studies which showed that arginine regulates the mTOR effectors in intestinal cells [38], [39]. This is either due to a cell line difference or to the arginine concentration that we used, which might not have been sufficient to trigger the signaling pathway. Furthermore, we measured surprisingly low values of TEER in cells cultivated with low arginine medium. This might be a direct consequence of the lower cell number or of maturational changes in tight junction proteins.

Taken together, we propose that the lack of arginine induced by mycoplasma infection is sensed by MDCK cells and triggers GCN2 pathway activation, which in turn results in exogenous gene reactivation.

## Supporting Information

Figure S1 **Effect of physiological amino acid levels on B^0^AT1-TMEM27 overexpressing MDCK cell cultures.** After viral transduction, B^0^AT1-TMEM27 overexpressing MDCK cells were subcultured on plastic dishes for 10 passages either in standard cell culture medium (CCAA) or in physiological medium (PAA). Western blotting experiments with antibodies directed against B^0^AT1 and TMEM27 were performed. The intensity of the immunoreactive bands was quantified, standardized to β-actin and normalized to CCAA. Data are represented as mean ± SEM (n = 3). No significance was observed when groups were compared by unpaired two-tailed t-test.
(TIF)

Figure S2 **Effect of physiological amino acid levels on expression of other transgenes.** A–B: Mycoplasma-infected MDCK cells overexpressing EGFP (A) or TMEM27 (B) were cultivated on filters in standard cell culture medium and treated for the indicated times with physiological medium. Quantitative RT-PCR analysis was performed and mRNA levels were standardized to 18S and normalized to time 0. Data are represented as mean ± SEM (n = 3). Groups were compared by one-way ANOVA followed by Dunnett post-test; *** p≤0.001.
(TIF)
