# Supplementary figures and images for: Amino Acids Regulate Transgene Expression in MDCK Cells
Source: PLoS One. 2014 May 5;9(5):e96823. doi: 10.1371/journal.pone.0096823 (PMC4010483; doi:10.1371/journal.pone.0096823)

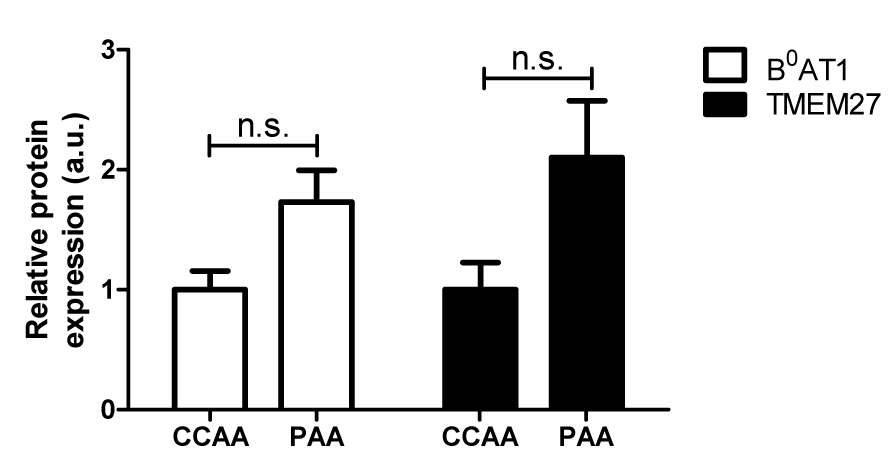

Supplement: Figure S1 — Effect of physiological amino acid levels on B0AT1-TMEM27 overexpressing MDCK cell cultures. After viral transduction, B0AT1-TMEM27 overexpressing MDCK cells were subcultured on plastic dishes for 10 passages either in standard cell culture medium (CCAA) or in physiological medium (PAA). Western blotting experiments with antibodies directed against B0AT1 and TMEM27 were performed. The intensity of the immunoreactive bands was quantified, standardized to β-actin and normalized to CCAA. Data are represented as mean ± SEM (n = 3). No significance was observed when groups were compared by unpaired two-tailed t-test. (TIF) [file pone.0096823.s001.tif]

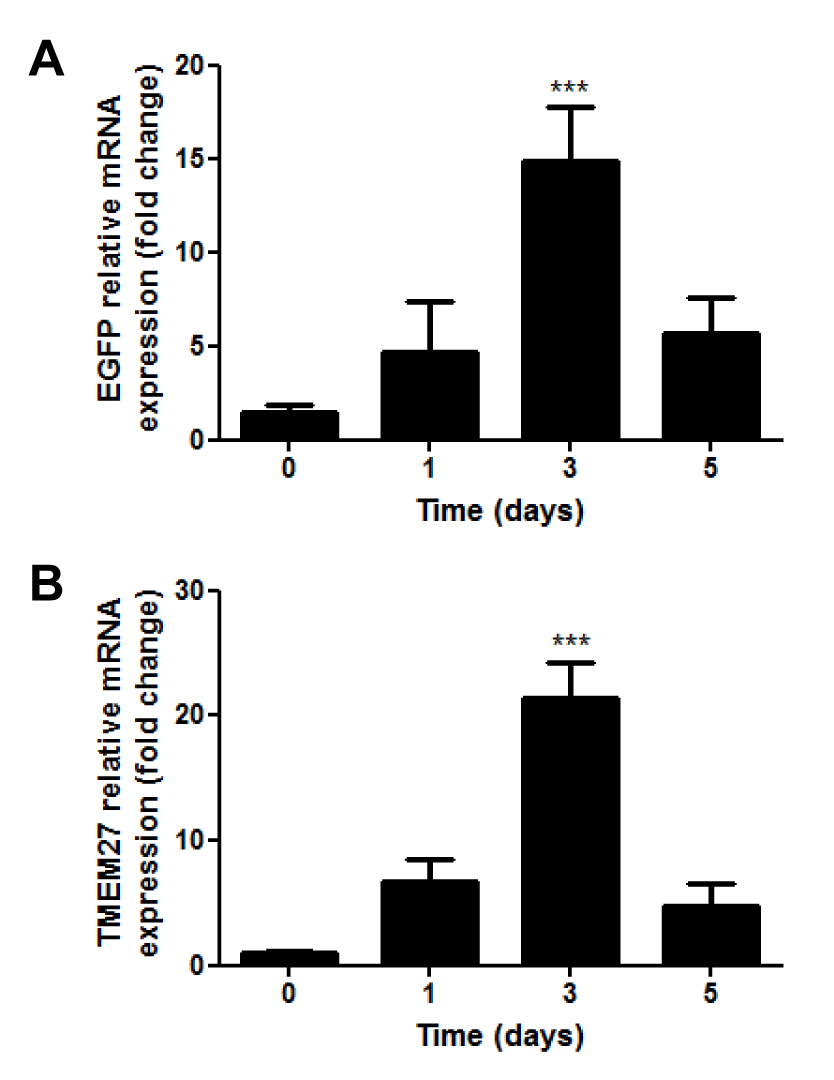

Supplement: Figure S2 — Effect of physiological amino acid levels on expression of other transgenes. A–B: Mycoplasma-infected MDCK cells overexpressing EGFP (A) or TMEM27 (B) were cultivated on filters in standard cell culture medium and treated for the indicated times with physiological medium. Quantitative RT-PCR analysis was performed and mRNA levels were standardized to 18S and normalized to time 0. Data are represented as mean ± SEM (n = 3). Groups were compared by one-way ANOVA followed by Dunnett post-test; *** p≤0.001. (TIF) [file pone.0096823.s002.tif]
